# A Primer on the Current State-of-the-Science Neoadjuvant and Adjuvant Therapy for Patients with Locally Advanced Rectal Adenocarcinomas

**DOI:** 10.1155/2012/863034

**Published:** 2012-05-16

**Authors:** Jeffrey T. Yorio, Nishin A. Bhadkamkar, Bryan K. Kee, Christopher R. Garrett

**Affiliations:** ^1^Unit 426, Department of Gastrointestinal Medical Oncology, The University of Texas MD Anderson Cancer Center, 1515 Holcombe Boulevard, Houston, TX 77030-4009, USA; ^2^Unit 462, Department of General Oncology, MD Anderson Cancer Center, 1515 Holcombe Boulevard, Houston, TX 77030-4009, USA

## Abstract

Patients with rectal cancers, due to the unique location of the tumor, have a recurrence pattern distinct from colon cancers. Advances in adjuvant therapy over the last three decades have played an important role in improving patient outcomes. This article serves to review the clinical studies that lay the basis for our current standard-of-care treatment of patients with locally advanced rectal cancer, as well as touch upon future ongoing experimental clinical trials of adjuvant chemoradiation therapy.

## 1. Introduction

Over one million people are diagnosed with colorectal cancer each year world-wide [1]. Patients with rectal cancers comprise approximately one-fifth of all patients with colorectal adenocarcinomas [2]; the unique anatomic location of the rectum with respect to the colon puts these patients at far higher risk of local recurrence [3]. Although adenocarcinoma is the most common cancer pathology of the rectum, squamous cell [4], adenosquamous [5], carcinoid cancers [6], and melanomas [7] also arise from the rectum, although with much lower prevalence. Anatomically the superior rectum is defined by the expansion of the taenia coli of the sigmoid colon to form a circular layer of muscle; inferiorly it is defined by the anorectal line (dentate line) [8]. The rectum is approximately 10–15 centimeters in length and endoscopically starts at 3 centimeters from the anal verge, extending to 15 centimeters, with significant person-to-person variation [9]. Patients with rectal cancer represent a subset of colorectal adenocarinoma patients that have been shown to have higher rates of recurrence after surgery alone when compared with more proximal portions of the colon secondary to its largely extraperitoneal situation [10]. Given the high rate of locally recurrent disease, multimodality therapy with a combination of total mesorectal excision (TME), radiation and chemotherapy (combined modality therapy, CMT) has now become the standard-of-care in locally advanced rectal cancer [11]. This paper seeks to review the seminal data that supports this approach, as well as touch on current controversies in the multimodality care of patients with locally advanced rectal cancer.

## 2. The Dawn of Adjuvant Therapy in Rectal Cancer

For many years, surgical resection was the only approach for patients with locally advanced rectal cancer. For patients with stage I disease, this continues to be the definitive treatment with a five-year overall survival (OS) rate of approximately 75% and a 7% or less local recurrence rate; however, patients with transmural penetration or nodal metastases have a higher risk of both local and distant recurrence, leading to inferior survival outcomes [12]. Given the high burden of local recurrence, efforts were initially placed into incorporating radiation therapy into the management of these patients as a means to improve local control. Chemotherapy was also incorporated into therapy to address potential micrometastatic disease (distant failure) as well as a tumor radiosensitizer [13].

In 1985, the Gastrointestinal Tumor Study Group (GITSG) published a randomized trial addressing the role of adjuvant radiation, chemotherapy, and chemoradiation in the treatment of locally advanced rectal cancer. A total of 227 patients were randomized after receiving surgical resection to four different groups including: (1) no adjuvant therapy, (2) adjuvant radiation alone at either 40 or 48 gray (Gy) dose, (3) adjuvant chemotherapy with semustine and 5-fluorouracil (5-FU), or (4) adjuvant combined modality therapy (CMT) with either 40 or 44 Gy radiation with concurrent 5-FU followed by post-radiation semustine plus 5-FU. At a median follow-up time of 80 months, patients in the control group had a local recurrence rate of 55% compared with only 33% in the adjuvant CMT arm. Additionally, progression-free survival (PFS) differed significantly amongst all four groups with the CMT arm being the most favorable (*P* < 0.04). In the initial report, there was a trend towards an OS benefit when comparing the control group to the CMT group (*P* = 0.07) [14]. In 1986, a follow-up report for this study showed that patients in the CMT group had a 24% estimated improved survival benefit at seven years (*P* = 0.005) [15].

After this study, there was still the question as to whether or not adjuvant CMT was truly superior to adjuvant radiation therapy alone. This was addressed in a prospective study of 204 post-operative patients with T3, T4 or node-positive rectal cancer who were randomly assigned to receive either adjuvant radiation or CMT. The adjuvant radiation arm was treated with 45 to 50.4 Gy, while the combined group received the same dosage of radiation with concurrent 5-FU. The CMT group was treated with one cycle of semustine-plus fluorouracil before and after radiation followed by an additional cycle of 5-FU. Patients in the radiation alone arm had an estimated five-year recurrence of 62.7% compared with 41.5% in the combination group. (*P* = 0.0016). More importantly, there was a 29% reduction in the overall death rate in the CMT group [11].

## 3. Chemotherapy versus CMT

As a result of these promising trials, the National Institute of Health (NIH) published a Consensus Statement in 1990 advocating the use of combined CMT for adjuvant treatment in stage II and III rectal cancers [17]. The National Surgical Adjuvant Breast and Bowel Project (NSABP) Protocol R-01 trial demonstrated that patients who received adjuvant radiation when compared to surgery alone had an overall reduction in local recurrence, but no difference in DFS and OS. Meanwhile, patients who received adjuvant chemotherapy had an improvement in DFS and OS when compared with patients who received only surgery [18]. Given these findings, the NSABP conducted a study, R-02, which randomized 694 patients to receive chemotherapy with or without radiation. Chemotherapy was given as either a regimen with bolus 5-FU modulated with leucovorin or with the MOF regimen which included semustine, vincristine, and 5-FU. Much like the NSABP Protocol R-01 trial, the addition of radiation to adjuvant chemotherapy did not improve disease-free survival (*P* = 0.90) or overall survival (*P* = 0.89) but did decrease the five-year incidence of local relapse from 13 percent to 8 percent (*P* = 0.02) [19].

## 4. Neoadjuvant Radiation

Since these trials were unable to demonstrate an OS benefit with postoperative radiation, many groups started to explore the use of radiation in the preoperative setting. In 1997, the Swedish Rectal Cancer Trial became the first trial to show survival benefit with the addition of preoperative neoadjuvant radiation [20]. In this trial, 1,168 patients were randomly assigned to receive either surgery alone or preoperative short-course radiotherapy followed by surgery. The radiation was given over 5 days for a total of 25 Gy within one week prior to surgical resection. Patients in the study in the radiation group had a much lower 5-year local recurrence rate of 11% versus 27% (*P* < 0.001). Meanwhile, the five-year survival rate was 58% in the radiation group compared with only 48% in the surgery alone group (*P* = 0.004) [21]. Long-term followup continued to show an OS benefit at 13 years [22].

Around the time that the Swedish Rectal Cancer Trial was being conducted, total mesorectal excision (TME) was being established as the gold-standard for surgical resection in rectal cancer [22]. The impressive results of TME called into question many of the previous neoadjuvant and adjuvant trials that did not utilize the optimal surgical method. The Dutch Colorectal Cancer Group (DCCG) addressed this by conducting a trial similar to the Swedish Rectal Cancer Trial comparing short-course radiotherapy (25 Gy over 5 fractions) with TME versus TME alone. At two years, the rates of local recurrence were 2.4% in the radiation group versus 8.2 percent in the TME only group (*P* < 0.001) [23]. At five years, local recurrence rates were 5.6 percent versus 10.9 percent (*P* < 0.001), but overall survival was only 64.2% in the radiation with TME group versus 63.5% in the TME only group (*P* = 0.902) [24].

## 5. Neoadjuvant CMT

Given the potential of neoadjuvant radiation and the prior success of postoperative chemoradiation, the next step in the treatment of locally advanced rectal adenocarcinoma was the exploration of neoadjuvant CMT in the preoperative setting [25]. The first of these was the German Rectal Cancer Study Group, which compared neoadjuvant CMT with adjuvant CMT in patients with T3, T4, or node-positive disease. The study enrolled 823 patients from 1995 to 2002. Patients assigned to the neoadjuvant CMT group received five weeks of preoperative chemoradiation (50.4 Gy given in 18 Gy per day over 28 fractions, five days per week). Patients were also given 5-FU as a protracted venous infusion at 1000 mg/m^2^ per day for five days on weeks one and five. A TME was then performed within six weeks of completion of neoadjuvant CMT. Patients in the adjuvant arm started four weeks after surgery and received the same schedule of CMT, with the exception of a 5.4 Gy boost. Both groups then received postoperative 5-FU at 500 mg/m^2^ per day for five days over four weeks. The study found no difference in 5-year OS between the two groups (76% for the neoadjuvant group and 74% for the adjuvant group, *P* = 0.8025). However, there was a lower local recurrence rate in the neoadjuvant group, 6% compared to 13% (*P* = 0.006). Additionally, it was found that the neoadjuvant group had significantly less long-term toxicities, particularly with regards to diarrhea, small bowel obstruction, and strictures at the anastomotic site [25].

The European Organization for Research and Treatment of Cancer (EORTC) published the results of Trial 22921 which also attempted to assess the addition of chemotherapy to preoperative radiotherapy. This trial randomized 1,011 patients to four different arms: (a) preoperative radiotherapy, (b) preoperative CMT, (c) preoperative radiotherapy with postoperative chemotherapy, or (d) preoperative CMT with postoperative chemotherapy. Radiation was given as 45 Gy delivered over 25 fractions; the 5-FU was given as a continuous infusion, modulated by leucovorin, for five days weeks one and five for the arms receiving preoperative CMT. Postoperative chemotherapy was given every three weeks for four cycles with the same regimen used preoperatively. The primary endpoint was OS between the two preoperative modalities and the two postoperative modalities. Ultimately, there was no difference in OS between the two groups that received preoperative radiation versus the two groups that received preoperative chemoradiation [26]. However, the group that did not receive any chemotherapy had a 5-year local recurrence of 17.1%. This was significantly higher than the preoperative CMT, the preoperative radiation with postoperative chemotherapy, and the preoperative CMT with postoperative chemotherapy groups, which had local recurrence rates of 8.7%, 9.6%, and 7.6%, respectively (*P* = 0.002) [26]. The trial was not designed to detect a difference in OS between the four groups, so this was not reported. Also of note, the trial ran for six years before it was required for patients to have a TME; thus, less than half of the patients were documented as having a TME.

The NSABP R-03 trial attempted to solidify the role neoadjuvant chemoradiotherapy as the treatment of choice for patients with stage II and III rectal cancer; in this study 267 patients were randomly assigned to either neoadjuvant CMT or adjuvant chemoradiation. Patients in the neoadjuvant group initially received a bolus of 5-FU with leucovorin once per week for six weeks. This was followed by radiation given as 45 Gy over 25 fractions with a 5.4 Gy boost. 5-FU and leucovorin were given on days 1–5 and days 21–25 of radiation. Patients then proceeded to surgery followed by 24 more weeks of weekly 5-FU and leucovorin. Patients in the adjuvant group followed the same treatment course with six weeks of chemotherapy, five weeks of CMT, and 24 weeks of chemotherapy all following initial surgery. Five-year disease-free survival (DFS) for the neoadjuvant group was 64.7 percent compared with 53.4 percent for the adjuvant group (*P* = 0.011) [27]. Additionally, there was observed a trend towards superior 5-year OS that was seen with 74.7% versus 65.6%, respectively (*P* = 0.65) [27]. Another interesting finding in this study was the 15% of patients in the neoadjuvant CMT group who obtained a complete pathologic response. In this small subset of patients, none of them had a recurrence. In this study it was not a requirement that all patients in this trial undergo a TME, which may have potentially confounded some of the results.

## 6. Optimizing Neoadjuvant Treatment

While questions still remain, for the most part, the results have established neoadjuvant CMT followed TME as the standard treatment in stage II and III rectal cancer with no contraindications to surgery or CMT. Subsequent trials have now tried to focus on optimizing both the length and types of chemotherapy and radiation used to improve survival and decrease toxicities.

### 6.1. Semustine

Many of the initial trials that favored adjuvant chemoradiation using 5-FU and semustine had concerns over the long-term toxic effects of semustine. In the first GITSG study [11], one patient who received semustine developed acute myelogenous leukemia (AML). The concerns over this toxicity led two trials to evaluate the benefit of adding semustine to 5-FU and radiation. Both studies found no differences in OS and semustine was ultimately excluded from future clinical studies.

### 6.2. 5-FU

The use of continuous infusion 5-FU over bolus 5-FU has become the standard of care in the perioperative treatment of rectal cancers primarily for its advantageous toxicity profile. In rectal cancer, the North Central Cancer Center Treatment Group (NCCTG) confirmed this by comparing adjuvant CMT with bolus 5-FU versus protracted venous infusion (PVI) [28]. Four-year relapse-free survival (RFS) was 53% in the bolus group and 63% in the continuous infusion group (*P* = 0.01), and four-year OS was 60% in the bolus group as compared with 70% in the continuous infusion group (*P* = 0.005) [28]. There was also significantly more diarrhea seen in the continuous infusion group versus more leucopenia in the bolus group.

### 6.3. Capecitabine

The backbone systemic therapy in CMT in the past has been 5-FU; while initially given as bolus therapy over 30 minutes, both prior to, with radiation, and following CMT, a randomized study demonstrated superiority of PVI 5-FU, in terms of decrease local relapse and improved OS [29]. Given the convenience of administration of the oral fluropyrimidines, and the fact that their administration which had similar pharmacokinetics to PVI 5-FU, capecitabine was studied in combination with radiation in the neoadjuvant CMT in rectal cancer patients. Phase I studies determined that the recommended phase II dose of capecitabine when combined with 50.4 Gy radiation preoperatively was 1800 mg/m^2^ daily given orally in two daily divided doses [30].

For most medical oncologists, capecitabine has become an acceptable equivalent alternative to 5-FU in the perioperative CMT treatment of rectal cancer. Much of this approach is extrapolated from the demonstrated efficacy of capecitabine in the adjuvant treatment of colon cancer [31]. Two randomized phase III studies evaluated the efficacy of capecitabine as a neoadjuvant radiosensitizing agent. The German trial compared the use of 5-FU to capecitabine in the perioperative CMT setting. Patients in the capecitabine arm received preoperative chemoradiation with 50.4 Gy and capecitabine 1,650 mg/m^2^ (in two divided doses) on days 1 through 38 plus capecitabine 2,500 mg/m^2^ days 1–14 every 21 days for five additional cycles. Patients were also assigned to receive the five additional cycles of capecitabine either before or after TME. Patients assigned to the 5-FU arm received neoadjuvant chemoradiation with 50.4 Gy and either 5-FU 225 mg/m^2^ daily or given as 1,000 mg/m^2^ on weeks one and five of radiation. Patients were also given four additional cycles of bolus 5-FU 500 mg/m^2^ for five days every 28 days. This was given either in the preoperative or postoperative setting. The five-year OS rate was 75.7% for the capecitabine group and 66.6% for the 5-FU group. This was significant for noninferiority (*P* = 0.0004) with a trend towards significance for superiority in favor of capecitabine (*P* = 0.053) [32].

A second randomized study, the NSABP R-04 trial, compared the use of capecitabine to continuous infusion 5-FU (both with or without oxaliplatin) during CMT. 5-FU was given as a 225 mg/m^2^ daily PVI during radiation and capecitabine was given at 1650 mg/m^2^ orally in two divided doses daily on the days of radiation only. No differences were seen with regards to pathologic complete response, surgical downstaging, or sphincter-saving surgery [33]. Local recurrence and overall survival have yet to be reported.

### 6.4. Oxaliplatin

Given the efficacy of oxaliplatin in the adjuvant [34] and metastatic [35] treatment of colon cancer, several recent trials have assessed the use of oxaliplatin in the perioperative treatment of rectal cancer. The Studio Terapia Adiuvante Retto (STAR)-01 trial has so far demonstrated a significant increase in toxicity, mainly diarrhea, without a benefit in local tumor response [36]. Similarly, the NSABP R-04 trial evaluated the addition of oxaliplatin with chemoradiation and found no improvement in pathologic complete response, surgical downstaging, or sphincter-saving surgery but did see a significant increase in grade 3 and 4 diarrhea (*P* < 0.0001) [33].

The German CAO/ARO/AIO-04 Trial showed that patients who received oxaliplatin with 5-FU during radiation had a pathologic complete response of 17.6% as compared with 13.1% (*P* = 0.033) for the group that received 5-FU alone during radiation [37]. The ACCORD 12/0405-Prodige Trial, which compared capecitabine with or without oxaliplatin during chemoradiation, demonstrated a similar statistical trend towards benefit with oxaliplatin, with pathologic complete response favoring the group receiving oxaliplatin, 13.9% compared to 19.2% (*P* = 0.09) [38]. Given no prospect clinical trial has demonstrated a survival advantage with the addition of oxaliplatin to CMT, preoperative oxaliplatin is currently not standard-of-care. Longer term followup for all of these studies is needed to evaluated DFS and OS before the efficacy of preoperative oxaliplatin can be assessed.

## 7. The Role of Additional Chemotherapy after Chemoradiation and Surgery

To date, there have not been any trials that have explored the use of further additional chemotherapy in rectal cancer after neoadjuvant chemotherapy and surgical resection. For the most part, medical oncologists use data from the adjuvant chemotherapy trials in stage II and III colon cancer as evidence and typically aim for a total of six months of perioperative treatment. The Multicenter International Study of Oxaliplatin/5-Fluorouracil/Leucovorin (FOLFOX) in the Adjuvant Treatment of Colon Cancer (MOSAIC) Investigators published the definitive trial that established the addition of oxaliplatin to 5-FU and leucovorin, the FOLFOX regimen, as the standard of care in the adjuvant setting [39]. Capecitabine with oxaliplain (CapeOx) has been shown to be superior to bolus 5-FU modulated by leucovorin (Mayo regimen) [16]. An equilvelance phase III study comparing FOLFOX with CapeOx is currently ongoing; safety data from this adjuvant trial suggest CapeOx is reasonably well tolerated [16]. It is generally recommended that patients with stage III or high-risk stage II colon cancer receive additional postoperative systemic fluropyrimidine-based adjuvant chemotherapy [11]. However, at this point, it is unknown whether or not patients with stage II rectal cancer truly benefit from additional adjuvant chemotherapy or if there is a subset of these patients who do not benefit from further treatment, similar to what is observed with the standard risk patients with stage II colon cancer.

## 8. Monoclonal Antibody Therapy in Neoadjuvant Treatment of Rectal Cancer

Monoclonal antibody therapies directed at circulating vascular endothelial growth factor (VEGF) and against cell receptor epidermal growth factor (EGFR) have become standard treatments in advanced colorectal cancer [40–42]. The anti-VEGF monoclonal antibody bevacizumab has been combined with capecitabine in a neoadjuvant CMT phase II single center study of 32 patients, with acceptable tolerance and a pathologic complete response rate of 32% [43]. Similar results were observed in another phase I/II single center trial, with promising clinical downstaging and a complete pathologic response rate of 23% (5 of 22 patients) [44]. Bevacizumab has also been combined with both oxaliplatin and capecitabine, in CMT; six of 25 (24%) patients achieved a complete pathologic response although there was noted to be significant gastrointestinal toxicity [45]. At this time no phase III trials evaluating the preoperative efficacy of bevacizumab are actively enrolling. Bevacizumab, cetuximab, and capecitabine are being combined with radiation preoperatively, in an ongoing current trial of KRAS non mutant rectal cancer patients [46]. The EXPERT-C trail was a randomized phase II study of preirradiation CAPOX followed by radiation therapy with capecitabine followed by TME, followed by postirradiation CAPOX; the experimental arm involved weekly concurrent monoclonal anti-EGFR therapy (cetuximab) administered with CAPOX. Of the 164 patients 90 (60%) were KRAS and BRAF non mutant. In this subset of patients the three-year OS was superior in the cetuximab-treated arm (96% versus 81%, *P* = 0.035), although there were no differences in the pathologic complete response rate [47].

## 9. Future Combined Modality Approaches to Locally Advanced Rectal Cancer

It is recognized that cancers in the upper one-third of the rectal have a lower risk of local recurrence when treated with surgery alone [48]; thus it is possible that some cancers, based on their anatomic location, may not benefit from the addition of radiation to chemotherapy and might be adequately treated with perioperative chemotherapy alone. However, this would have to be confirmed by randomized clinical trials before altering the current standard-of-care. A four-stage combined modality approach (chemotherapy, chemoradiation, surgery, and postoperative chemotherapy), as demonstrated by the EXPERT-C trial referenced above [47], is also being actively evaluated. The duration of preoperative chemotherapy is also being addressed in studies; a three arm trial of chemoradiation, versus chemoradiation and two cycles FOLFOX chemotherapy, versus chemoradiation and 4 cycles FOLFOX chemotherapy, demonstrated a higher pathologic complete response rate associated with the more preoperative FOLFOX chemotherapy, without increasing the surgical complication rates [49]. Whether or not this approach will lead to higher OS rates is currently unclear.

## 10. Conclusions

The multidisciplinary management of rectal cancer, with the incorporation of radiation and chemotherapy into the treatment plan, has had a significant impact on survival outcomes. Future approaches will likely tailor therapies and approaches based upon the anatomic location of the tumor, the molecular features, and possibly the pathologic response to neoadjuvant therapy. While 5-FU and capecitabine remain the standard therapy for combination with radiation, future studies may define a role for subsets of patients who benefit from the addition of oxaliplatin and 5-FU or capecitabine combined with radiation. The optimal preoperative dose of radiation, treatment schedule, and type of radiation treatment planning techniques continue to be evaluated prospectively in clinical trials. Future significant advances in systemic therapies hold the prospect of decreasing the necessity of surgery or radiation in rectal cancer.

## References

[1] Jemal A, Bray F, Center MM, Ferlay J, Ward E, Forman D (2011). Global cancer statistics. *CA Cancer Journal for Clinicians*.

[2] Siegel R, Naishadham D, Jemal A (2012). Cancer statistics, 2012. *CA Cancer Journal for Clinicians*.

[3] Rosen L, Veidenheimer MC, Coller JA, Corman ML (1982). Mortality, morbidity, and patterns of recurrence after abdominoperineal resection for cancer of the rectum. *Diseases of the Colon and Rectum*.

[4] Landau D, Garrett C, Chodkiewicz C (2007). A case of primary squamous cell colon cancer. *Journal of Oncology Pharmacy Practice*.

[5] Dong Y, Wang J, Ma H, Zhou H, Lu G, Zhou X (2009). Primary adenosquamous carcinoma of the colon: report of five cases. *Surgery Today*.

[6] Koura AN, Giacco GG, Curley SA, Skibber JM, Feig BW, Ellis LM (1997). Carcinoid tumors of the rectum: effect of size, histopathology, and surgical treatment on metastasis free survival. *Cancer*.

[7] Homsi J, Garrett C (2007). Melanoma of the anal canal: a case series. *Diseases of the Colon and Rectum*.

[8] Morgan CN (1936). The surgical anatomy of the anal canal and rectum. *Postgraduate Medical Journal*.

[9] Memon S, Keating JP, Cooke HS, Dennett ER (2009). A study into external rectal anatomy: improving patient selection for radiotherapy for rectal cancer. *Diseases of the Colon and Rectum*.

[10] Cass AW, Million RR, Pfaff WW (1976). Patterns of recurrence following surgery alone for adenocarcinoma of the colon and rectum. *Cancer*.

[11] Engstrom PF, Arnoletti JP, Benson AB (2010). Anal carcinoma: clinical practice guidelines in oncology: rectal cancer. *Journal of the National Comprehensive Cancer Network*.

[12] Gunderson LL, Sargent DJ, Tepper JE (2004). Impact of t and n stage and treatment on survival and relapse in adjuvant rectal cancer: a pooled analysis. *Journal of Clinical Oncology*.

[13] McGinn CJ, Kinsella TJ (1993). The clinical rationale for S-phase radiosensitization in human tumors. *Current Problems in Cancer*.

[14] Gastrointestinal Tumor Study Group (1985). Prolongation of the disease-free interval in surgically treated rectal carcinoma. *The New England Journal of Medicine*.

[15] Douglas HO, Moertel CG, Mayer RJ (1986). Survival after postoperative combination treatment of rectal cancer. *The New England Journal of Medicine*.

[16] Haller DG, Tabernero J, Maroun J (2011). Capecitabine plus oxaliplatin compared with fluorouracil and folinic acid as adjuvant therapy for stage III colon cancer. *Journal of Clinical Oncology*.

[17] Krook JE, Moertel CG, Gunderson LL (1991). Effective surgical adjuvant therapy for high-risk rectal carcinoma. *The New England Journal of Medicine*.

[18] Hall WH (1990). Adjuvant therapy for patients with colon and rectal cancer. *JAMA*.

[19] Fisher B, Wolmark N, Rockette H (1988). Postoperative adjuvant chemotherapy or radiation therapy for rectal cancer: results from nsabp protocol R-01. *Journal of the National Cancer Institute*.

[20] Wolmark N, Wieand HS, Hyams DM (2000). Randomized trial of postoperative adjuvant chemotherapy with or without radiotherapy for carcinoma of the rectum: national surgical adjuvant breast and bowel project protocol R-02. *Journal of the National Cancer Institute*.

[21] Påhlman L (1997). Improved survival with preoperative radiotherapy in resectable rectal cancer. *The New England Journal of Medicine*.

[22] Folkesson J, Birgisson H, Pahlman L, Cedermark B, Glimelius B, Gunnarsson U (2005). Swedish rectal cancer trial: long lasting benefits from radiotherapy on survival and local recurrence rate. *Journal of Clinical Oncology*.

[23] MacFarlane JK, Ryall RDH, Heald RJ (1993). Mesorectal excision for rectal cancer. *The Lancet*.

[24] Kapiteijn E, Marijnen CAM, Nagtegaal ID (2001). Preoperative radiotherapy combined with total mesorectal excision for resectable rectal cancer. *The New England Journal of Medicine*.

[25] Peeters KCMJ, Marijnen CAM, Nagtegaal ID (2007). The tme trial after a median follow-up of 6 years: increased local control but no survival benefit in irradiated patients with resectable rectal carcinoma. *Annals of Surgery*.

[26] Sauer R, Becker H, Hohenberger W (2004). Preoperative versus postoperative chemoradiotherapy for rectal cancer. *The New England Journal of Medicine*.

[27] Bosset JF, Collette L, Calais G (2006). Chemotherapy with preoperative radiotherapy in rectal cancer. *The New England Journal of Medicine*.

[28] O’Connell MJ, Martenson JA, Wieand HS (1994). Improving adjuvant therapy for rectal cancer by combining protracted- infusion fluorouracil with radiation therapy after curative surgery. *The New England Journal of Medicine*.

[29] Roh MS, Colangelo LH, O’Connell MJ (2009). Preoperative multimodality therapy improves disease-free survival in patients with carcinoma of the rectum: NSABP R-03. *Journal of Clinical Oncology*.

[30] Ngan SYK, Michael M, Mackay J (2004). A phase i trial of preoperative radiotherapy and capecitabine for locally advanced, potentially resectable rectal cancer. *British Journal of Cancer*.

[31] Twelves C, Wong A, Nowacki MP (2005). Capecitabine as adjuvant treatment for stage III colon cancer. *The New England Journal of Medicine*.

[32] Hofheinz R, Wenz FK, Post S (2011). Capecitabine versus 5-fluorouracil-based (neo)adjuvant chemoradiotherapy for locally advanced rectal cancer: long-term results of a randomized, phase III trial. *Journal of Clinical Oncology*.

[33] Roh MS, Yothers GA, O'Connell MJ (2011). The impact of capecitabine and oxaliplatin in the preoperative multimodality treatment in patients with carcinoma of the rectum: NSABP R-04. *Journal of Clinical Oncology*.

[34] André T, Boni C, Mounedji-Boudiaf L (2004). Oxaliplatin, fluorouracil, and leucovorin as adjuvant treatment for colon cancer. *The New England Journal of Medicine*.

[35] Goldberg RM, Sargent DJ, Morton RF (2004). A randomized controlled trial of fluorouracil plus leucovorin, irinotecan, and oxaliplatin combinations in patients with previously untreated metastatic colorectal cancer. *Journal of Clinical Oncology*.

[36] Aschele C, Pinto C, Cordio S (2009). Final safety findings from a randomized phase III trial of preoperative FU-based chemoradiation +/- weekly oxaliplatin as neoadjuvant therapy for patients with locally advanced rectal cancer: the STAR (Studio Terapia Adiuvante Retto)-01 randomized trial. *Gastrointestinal Cancers Symposium*.

[37] Roedel C, Becker H, Fietkau R (2011). Preoperative chemoradiotherapy and postoperative chemotherapy with 5-fluorouracil and oxaliplatin versus 5-fluorouracil alone in locally advanced rectal cancer: first results of the German CAO/ARO/AIO-04 randomized phase III trial. *Journal of Clinical Oncology*.

[38] Gérard JP, Azria D, Gourgou-Bourgade S (2010). Comparison of two neoadjuvant chemoradiotherapy regimens for locally advanced rectal cancer: results of the phase iii trial accord 12/0405-prodige 2. *Journal of Clinical Oncology*.

[39] André T, Boni C, Navarro M (2009). Improved overall survival with oxaliplatin, fluorouracil, and leucovorin as adjuvant treatment in stage II or III colon cancer in the mosaic trial. *Journal of Clinical Oncology*.

[40] Koukourakis GV, Sotiropoulou-Lontou A (2011). Targeted therapy with bevacizumab (Avastin) for metastatic colorectal cancer. *Clinical and Translational Oncology*.

[41] Hoda D, Simon GR, Garrett CR (2008). Targeting colorectal cancer with anti-epidermal growth factor receptor antibodies: focus on panitumumab. *Therapeutics and Clinical Risk Management*.

[42] Garrett CR, Eng C (2011). Cetuximab in the treatment of patients with colorectal cancer. *Expert Opinion on Biological Therapy*.

[43] Crane CH, Eng C, Feig BW (2008). Phase II trial of neoadjuvant bevacizumab, capecitabine, and radiotherapy for locally advanced rectal cancer. *Journal of Clinical Oncology*.

[44] Willett C, Duda D, Boucher Y (2007). Phase III study of neoadjuvant bevacizumab with radiation therapy and 5-fluorouracil in patients with rectal cancer: initial results. *Journal of Clinical Oncology*.

[45] Dipetrillo T, Pricolo V, Lagares-Garcia J (2009). Neoadjuvant bevacizumab, oxaliplatin, 5-fluorouracil, and radiation in clinical stage II-III rectal cancer. *Journal of Clinical Oncology*.

[46] Elvira G, Torrecillas L, Cervantes G (2011). Phase II study of bevacizumab and cetuximab as neoadjuvant treatment in locally advanced rectal cancer: a preliminary security report. *Journal of Clinical Oncology*.

[47] Dewdney A, Capdevila J, Glimelius B (2011). EXPERT-C: a randomized, phase II European multicenter trial of neoadjuvant capecitabine plus oxaliplatin chemotherapy and chemoradiation with or without cetuximab followed by total mesorectal excision in patients with MRI-defined, high-risk rectal cancer. *Journal of Clinical Oncology*.

[48] Nash GM, Weiss A, Dasgupta R, Gonen M, Guillem JG, Wong WD (2010). Close distal margin and rectal cancer recurrence after sphincter-preserving rectal resection. *Diseases of the Colon and Rectum*.

[49] Garcia-Aguilar J, Marcet J, Coutsoftides T (2011). Impact of neoadjuvant chemotherapy following chemoradiation on tumor response, adverse events, and surgical complications in patients with advanced rectal cancer treated with total mesorectal excision. *Journal of Clinical Oncology*.

